# Deepening Context in Realist Evaluation: Exploring Adolescent Agency in Sexual and Reproductive Health in Urban India

**DOI:** 10.34172/ijhpm.8837

**Published:** 2026-06-10

**Authors:** Sara Van Belle, Sana Contractor, Sandhya Gautam

**Affiliations:** ^1^Department of Public Health, Institute of Tropical Medicine, Antwerp, Belgium.; ^2^Independent Consultant, Mumbai, India.; ^3^Seher - Center for Health and Social Justice, New Delhi, India.

**Keywords:** Realist Evaluation, Adolescent, Accountability, Sexual and Reproductive Health, Agency and Empowerment, India

## Abstract

**Background:**

Although accountability interventions to strengthen adolescent sexual and reproductive health (ASRH) service delivery in marginalized urban neighbourhoods are on the rise, evidence on the underlying mechanisms and context conditions is scarce. We analysed the strategies of two grassroots organizations in India that support adolescent agency in sexual and reproductive health (SRHR) to identify the mechanisms and context conditions in which they work.

**Methods:**

We used realist evaluation (RE) and a case study design with two sites in Delhi and Mumbai. We used Margaret Archer’s structure-agency-culture (SAC) framework to deepen the analysis of context and of the temporal dynamics of the intervention.

**Results:**

We found that the organizations used five strategies: (1) mobilization of girls and young women, (2) raising critical consciousness, (3) supporting self-organization and emergent collective action, (4) documenting lived realities and engagement with local governance actors. Organizational and relational mechanisms triggered include trust-building in the community and with parents, which enables support, fosters a group identity, and provides a safe space for peer exchange. Enhancing reflexivity, self-efficacy and a sense of place among adolescent girls, the intervention stimulates emergent collective action within the neighbourhood, which in turn contributes to build consensus and enforce accountability with local and sub-national governance actors. We considered causal mechanisms to be historically grounded in practices preceding current ones, and we adopted a view on "context" that includes a longer-term temporal dimension.

**Conclusion:**

The refined programme theory confirms that organizations will simultaneously engage in consensus-oriented and agonistic strategies with state actors in their efforts to achieve social change.

## Background

**Key Messages**

**Implications for policy makers**

- Policy-makers should engage pro-actively with adolescents to support their health and well-being, and with the grassroots organizations and communities working alongside them.
- Grassroots organizations demonstrate nimbleness and agility in their efforts to influence local public policy decisions concerning adolescent health.
- Power dynamics within the local governance system, and the power of local countervailing norms, should not be underestimated by policy-makers and stakeholders at other levels seeking to implement adolescent health interventions.

**Implications for the public**

Broader gender-based social norms and contexts have a deep impact on the health of girls and young women as well as their ability to utilize benefits offered by government schemes and services. This evaluation study shows that feminist grassroots organizations in India play an important role in bringing about social change, through fostering collective action, empowerment and autonomy of adolescent girls and young women. In order to be successful, their efforts require the support of local communities and parents who also need to be actively engaged to support the inclusion of adolescent and young women’s voices in local decision-making.

Shaped by many intersecting factors that lead to high (sexual and reproductive) health risks, the health status of many adolescent girls and young women living in marginalized urban neighbourhoods is precarious. Social and neighbourhood attributes, such as access to housing, livelihood, safety, concentrated poverty and social exclusion, are important determinants for sexual and reproductive health (SRHR). Access to SRHR information is inadequate and where available, it is often unreliable. When public services are available, their accessibility is marred by concerns around mobility, stigma and provider bias. Yet, the urban environment can also present a space for adolescent agency and empowerment, when local roots organizations work with stakeholders to strengthen the voice of adolescents to achieve better health services. Cities can be spaces for effective collective and individual agency of adolescents.

 Adolescents are considered ‘the missing population’ in terms of strengthening health systems to achieve universal health coverage.^1^ Both demand and supply side interventions for adolescent-friendly service delivery have been explored from this perspective. Often, these focus on programmatic strategies, such as community scorecards on the demand side, or training of healthcare providers in respectful care on the supply side.Strategies to improve access to adolescent sexual and reproductive health (ASRH) services include adolescent-friendly corners in health facilities or specific consultation hours. In India, where this study is located, government has broadened its interest in young people and adolescent health, from focusing narrowly on early marriage, nutrition and reproductive health, to addressing the health of this group more holistically. In order to strengthen preventive, diagnostic, and curative services throughout the health system and to promote adolescent health through a community-based approach, the Ministry of Health and Family Welfare introduced Rashtriya Kishor/i Swasthya Karyakram in 2014. It envisages the setting up of adolescent-friendly health clinics and community-based services, and organising adolescent health days. Non-governmental organizations (NGOs) working with youth have been engaging with these programs.

 Globally, standalone interventions are increasingly abandoned and replaced by integrated approaches.^2^ The need for stakeholder engagement and cocreation in such approaches, including giving youth a voice and supporting the development of civic engagement of youth, is increasingly felt. It is emerging on the global agenda in the form of agency-supporting interventions, participation in decision-making of youth-serving organizations, and engagement of adolescents in co-creation of research and policy. This can enhance supply side interventions by increasing the voice and agency of adolescents and by including them in decision-making.^3,4^ Improved stakeholder engagement can also leverage local collaborative governance in support of adolescent-friendly public services.^5^ Flores et al suggest that grassroots organizations can play a critical role in ensuring local public accountability.^6^ We define grassroots organizations as “*groups of people pursuing common interests, largely on a volunteer and not-for-profit basis. Often such organizations are formed by activists in social movements. Many are closely linked to communities and local concerns. The term often refers to voluntary associations through which ‘disadvantaged’ people organise themselves to improve the social, cultural, and economic wellbeing of their families, communities, and societies.*”^7^

 Yet, adolescent agency and voice in decision-making in health remains under-explored and under-theorized.^8,9^ There is also little evidence regarding “context,” both in its spatial and temporal dimensions. First, not much is known on the “sense of place” of adolescents – the meaning they attach to living in a particular urban (marginalized) neighbourhood and how that shapes their choices and behaviour in health-related matters.^10^ Second, the local accountability and governance ecosystem in which externally funded interventions to strengthen adolescent-responsive services are embedded is hardly examined.^11^

 This paper seeks to build an understanding of how such interventions “work” using realist evaluation (RE)^12,13^ through a multiple case study design^14^ in two marginalized urban neighbourhoods in Delhi and Mumbai, India. The central research question was: *Why, how and in which conditions can organizational strategies of youth-serving organizations in poor marginalized neighbourhoods contribute to improved adolescent health and well-being? *We explored the strategies of two grassroots organizations to better understand if and how they strengthen the voice and agency of adolescents in local governance. Further, we sought to deepen the understanding of context, and the role it plays in how interventions work. While RE explicitly considers the role of context in the analysis, guidance on how to identify context factors and analyse their role in the causal configuration is not well developed yet. In this study, we use Margaret Archer’s structure-agency-culture (SAC) framework, which emphasizes the roles of structure (social political organizations, institutions), agency (capacity of individuals and groups to take action) and culture (beliefs, social norms) in bringing about change. This allows not only to focus the question of adolescent agency, which the interventions seek to enhance, but also to unpack context into cultural and structural factors and take into account the temporal dimensions relating to long-standing grassroots interventions.

## Methods

 RE is well-suited to study complex social phenomena.^12^ Central to RE is the programme theory, or the hypothesis on how a situation or an outcome of interest is the result of mechanisms that shape the actions of actors in specific context conditions. Realist researchers aim at developing, empirically testing and refining these programme theories. The generative causality perspective of RE brings the role of context to the fore. Realists postulate that causal mechanisms underlie human action and that these are triggered in specific contexts. They use a causal configurational heuristic, the context-mechanism-outcome (CMO) configuration^12^ (as used in this paper) or an adapted version, like the intervention-context-mechanism-outcome configuration^15^ to analyse the data. The CMOs are the key elements for building the initial programme theory (IPT) at the start of the realist cycle and to refine the programme theory at the end of empirical studies.

 In a previous publication, we developed methodological guidance to deepen the realist analysis of context based on the SAC framework of Margaret Archer. We showed how the SAC framework can contribute to developing the IPT by introducing a time-sensitive context analysis that allows for explaining emergence as central to social change.^16^ In that paper, we presented the IPT, which we tested in the India case studies presented below.

 We structured our study following the realist research cycle,^17^ which we adapted to include the SAC framework. It has four steps: (1) to elicit the IPT; (2) to empirically test the IPT in two study sites; (3) to refine the IPT and (4) to draw cross-case lessons. This approach is intended to aid realist theorizing.^18^ All along the study development and implementation, we followed the guidelines for RE.^19^

###  Study Design 

 We adopted a multiple case study design.^14^ We defined the case as “organizational accountability strategies to enhance the agency and SRHR and well-being outcomes of adolescent girls.” We purposively selected two marginalized urban neighbourhoods in major cities (New Delhi and Mumbai) as the study sites. In each site, we selected a grassroots organization working with adolescent girls with intersecting vulnerabilities. The organizations were chosen based on the following criteria: (1) their rights-based approach to empowerment of youth, rooted in feminist principles, (2) the diversity of their strategies of engagement with youth and state actors, and (3) previous engagement of the researchers with the organizations, and the resulting trust and rapport with the communities and respondents. To enhance confidentiality, we refer in the text below to the study sites as neighbourhood 1 and 2 (N1&2) and organization 1 and 2 (O1&2). The overall objectives of the study and the themes of the interviews were discussed with the leaders of the grassroots organizations at the very start of the study. This led to minor modifications of the themes and questions.

###  Data Collection

 RE is essentially method-neutral: the researcher needs to collect the data required to test the IPT.^17^ We carried out a document review, in-depth interviews (IDIs) and focus group discussions (FGDs). The data collection was conducted by the SQC and SG between July and December 2021.

 The document review covered publications, annual reports, project reports, newsletters, and manuals of the organizations. We also scanned websites, publications and media reports. This allowed us to describe the organization’s policies, background and context.

 Nine FGDs, each including eight participants, were held: five in N1 and four in N2. The respondents were adolescents (15 to 17 years) and young women (18 to 25 years), all involved with the organization’s programmes. The duration of the FDGs was 45-75 minutes.

 We carried out 21 IDIs: seven with staff of the organizations, three with staff of other NGOs working on gender and accountability in the same neighbourhood, two with elected representatives and nine with service providers (teachers and doctors) and community health workers (Accredited Social Health Activists, India). We used an interview guide based on the IPT. The FGD and interview guides are available as Supplementary files 1-4.

 The selection of respondents was purposive, aimed at including a wide diversity of views. Discussions were held with field staff of both organizations, emphasizing the importance of obtaining diverse perspectives in order to draw lessons from their experience. This created a favourable environment and allowed the respondents to express adverse experiences or share disappointments, which could be explored further. The final selection of participants was done in consultation with the organization, and one fieldworker accompanied the researchers while recruiting. None of the persons we approached declined participation, but it should be noted that two governance actors in the site of O1 and one in O2 hesitated in giving us appointments. After follow up, we decided not to press further for these interviews.

 The interviews and FGDs were recorded after informed consent was obtained. They were transcribed and translated verbatim by research assistants. Translation and transcription checks were carried out by authors SG and SQC on each other’s data. Both authors are native speakers of the language in which the data collection was carried out. Audio recordings were erased after transcription and pseudonymisation, in conformance with ethics requirements. The data was entered and coded in QDA Miner Lite (v. 2.0.9).

###  Data Analysis

 The analysis started with drafting a thick description of each study site,^20^ including the context, the organization, its policies, and its activities. These were presented to representatives of the organizations for respondent validation. Subsequently, a realist analysis of the interviews and FGD was conducted following the guidance presented by Van Belle et al.^16^ In a first coding of the transcripts, we used a coding tree drawn from the IPT, to which emerging codes were added as needed. In the second round, causal relationships between organizational strategies, structural and cultural context conditions, the agency of the actors and underlying mechanisms were examined. In the Results section, we describe these steps in detail.

###  Analytic Rigor and Positionality

 In line with realist principles, we did not seek to eliminate researcher bias but instead to reflect on and be aware of our positionality, and strengthen the credibility of our research findings through periodic checks. Because the two embedded researchers had prior deep relationships with the organizations, they were able through their rapport with the participants to ensure that the interpretations of the data were vetted and thus accurate. Regular meetings within the research team provided direction for triangulation of data with other informants or with documents, and allowed for checking when there were inconsistencies. This reflexive and iterative process allowed us to obtain a layered understanding of the specific context of each case. Each stage of coding (described above) was discussed between the three researchers as well as research assistants and one person from the organization, to ensure validation in the coding process. Disagreements were discussed by the research team and consensus was sought.

###  Reproducibility and Generalizability

 Philosophically, our research approach does not guarantee reproducibility in the way that a laboratory experiment or trial would. This study presents a pattern that may not be generalizable – RE is about deepening context specificity and *plausible *causal explanations.^21^ Owing to this, interventions in other cities of India, (in other regions of the country or tier 2 or 3 cities) which have different histories, socio-cultural contexts and governance structures, may produce different outcomes.

###  Ethics Statement 

 The study protocol was approved by the Institutional Review Board of the Centre for Health and Social Justice, New Delhi. We applied the Indian Council of Medical Research guidelines on ethical research in health involving human participants^22^ and involving children^23^ and the World Health Organization (WHO) guidelines for research on sexual and reproductive health with adolescents.^24^ We acknowledged the vulnerability of the adolescents and young women of urban poor neighbourhoods who are engaged in the activities of the grassroots organizations.^25^ We focused on cultural sensitivity in our data collection, aligning with prevailing cultural norms. The research team included members who lived close to the research site and have worked with adolescent girls before. Both were familiar with local social norms, as well as language/terms that the respondents used.

###  Positionality Statement

 The authorship team consists of three researchers, of which one (based in Europe) acquired funding and the other two were embedded in the cities and had a prior relationship with the organizations where the study was conducted. The third researcher discussed the interpretation of data with the embedded researchers but adhered in the analysis to the embedded researchers’ interpretation. Any existing power asymmetries related to funding of the study (one researcher acquiring funding) were buttressed by the acknowledged deeper contextual knowledge of the two researchers, the common interest and passion for the subject of study, confirmed by a long-term engagement of two members in a global network. Power asymmetries also exist between the embedded researchers and the organizations through whom the study was conducted. We attempted to reduce the impact of this by ensuring that the process and findings of the study were discussed and co-created at different stages, emphasizing the learning agenda of the study.

## Results

 Below, we present the results, illustrating how we applied the methodological guidance presented by Van Belle et al on the use of the SAC framework in this realist study.^16^

###  Step 1: Information Gleaning on Context

 We first present the findings related to the neighbourhood’s context and the grassroots organizations, which provides the background for the analysis in Step 3.

 In Indian cities, the Municipal Corporation (municipal authorities) and the State Government are responsible for the delivery of public services, including water, sanitation and most of the healthcare and education services. In both neighbourhoods, the delivery of public services is inadequate. N1 is an area where mostly long-term migrants reside. Families are employed in the informal sector, making clothes, toys or slippers. People live in small two-storied tenements, separated by narrow lanes with open drainage channels. The water supply is erratic as water pipes are often broken and there is a dearth of toilets, although many households have a public toilet nearby.

 Migrants of four generations live in N2. Women are mostly at home and men work in the informal sector. Disregard for norms of construction resulted in unsafe housing, lack of proper water supply, electricity and sewerage and overpopulation. Although most houses have electricity, in some cases illegally. The municipality provides water once or twice a day to households without water supply. The relationship between the community and state actors is tenuous, as this marginalized neighbourhood has been stereotyped a “hotbed of terror,” and subjected to surveillance operations.

 O1 has its origins in the Indian feminist movement of the 1980s, and more specifically in the broad social mobilization against domestic violence and the burning of brides. Its modus operandi consisted initially of supporting women’s collectives to improve services in their communities for various issues. Later, they worked with adolescent girls on health, which was approached from a holistic and gender-sensitive perspective instead of the common programmatic focus on reproductive healthcare. The organization consistently focused on strengthening grassroots leadership in collective action and on emerging issues relevant to the community. The latter included violence against women and self-help between 1980-2005, promoting the education of girls, and water and sanitation problems (2007), menstrual health and hygiene (2009), women’s public safety in urban areas (2015) and environmental issues (2019).

 O2 was established in 2012 by local professional women who were active in another NGO working on the marginalization of Muslim women since 1987. O2 focuses on youth community football for girls, which is intended to empower adolescent girls in public spaces. Alongside community sports, the girls are educated on gender issues. O2 addresses emergent issues with the girls in the neighbourhood, such as mental health during the COVID-19 pandemic and creation of jobs. Since 2012, O2 reached 200 girls. The coaches for the football teams are earlier members, strengthening the girls’ leadership capabilities. O2 attracted attention from policy-makers who want to be associated with its work and has sought collaboration from the state government for organising public events.

 Both organizations use a combination of strategies to support adolescent girls’ agency and bring about local social change.

###  Step 2: Developing Context-Mechanism-Outcome Configurations

 We found that the organizations used five strategies: (1) mobilization of girls and young women, (2) raising critical consciousness, (3) supporting self-organization and emergent collective action, (4) documenting lived realities and engagement with local governance actors. For each strategy, we present the summary of CMO configurations below.

####  Context-Mechanism-Outcome Mobilisation of Girls and Young Women 

 We found that parents and community members consider extracurricular activities of adolescent girls unnecessary and that they fear this will lead girls astray. The organizations thus must obtain support from the adolescent girls’ parents. To this end, O2 organises home and school visits.

 “*I think that our relationships with the girls’ family members are very important. (…) I believe that one should make a strong relationship with at least one member of the family (…). If you get that support, you can do anything” *(O2, staff).

 The organizations build on the trust of the community and families and on feelings of solidarity. The engagement of the mothers is important, which is easier to obtain when they are already organized, as is the case in N1. O2 sometimes acts as mediator with older brothers who often act as proxy head of household and have a great influence on what adolescent girls can and cannot do (See Table 1).

**Table 1:** Context-Mechnism-Outcome Configuration Mobilisation of Girls and Young Women

**Organizational strategy**	**CMO Configuration**	**Organization **
Mobilisation of girls and young women	Grassroots organizations originating from or embedded in local communities mobilise adolescent girls and young women (O) through home visits or school visits (I). Support of parents is more likely if mothers are already member of the organization (C).	O1 and O2
	In a context of limited opportunities (C), adolescent girls and their parents join the organization because of perceived gains in the form of opportunities for training and opening doors to other opportunities (M), trust in the organization (M) or because of neighbourhood solidarity (M).	O2

Abbreviation: CMO, context-mechnism-outcome.

####  Context-Mechnism-Outcome Raising Critical Consciousness

 The organizations raise critical awareness of adolescent girls and young women mainly through training. The topics include gender issues and rights; body awareness; nutrition; menstrual, SRHR; life skills (such as negotiating family and love relationships, friendship, setting life goals); and government laws, policies, programmes, and schemes.

 The girls are also taught how to manage and be empowered in relationships:

 “*We want girls to be assertive. Even when they are compromising, they must know that they are compromising. All the girls must be in relationships with some awareness about what is right and what is not” (O2, staff member).*

 O2 uses football as a platform and “field” for learning. They help girls reflect on their behaviour, aggression, collaboration, team building and competitiveness through the metaphor of sport. Football is also used as a way to build the self-confidence of girls and question stereotypes of good behaviour of women (See Table 2):

**Table 2:** Context-Mechnism-Outcome Configuration Raising Critical Consciousness

**Organizational strategy**	**CMO Configuration**	**Organization **
Raising critical consciousness	In a context where girls and young women have lit-tle decision-making power, owing to their age, gen-der and social marginalization (C), educational group activities focusing on their body experience, SRHR entitlements, and sports activities transcend-ing traditional gender roles (I) empowers girls as individual and collective agents (O) and leads to an increased ability to make decisions (O). This pro-cess of individual empowerment is grounded in en-hanced reflexivity (M) and self-efficacy (belief in one’s ability to take the right decisions) (M).	O1 and O2

Abbreviations: CMO, context-mechnism-outcome; SRHR, sexual and reproductive health.

 “*Football was never an end for us. Football is a strategy of perspective building. If you are able to come out and play, then the confidence that you develop gets carried forward to so many places. So, we don’t just do football training, we do perspective sessions along with it, too. Our goal is very clear - that all our girls are able to earn money. Self-respect is very important and you should never have to be dependent on a man. Football helps us with that” *(O2, staff member).

 “*We used to feel so powerful and felt like we could do anything” *(Adolescent girl member, O2).

####  Context-Mechnism-Outcome Supporting Self-organization and Emergent Collective Action 

 Both organizations stimulate self-organization of adolescents and young women and collective action through various ways. Joint sense-making is a central theme. The organizations set up small groups of girls and women to share experiences. Discussing their problems (for instance domestic violence) and critical events allows the girls to exchange views and perspectives, and to support each other, which triggers reciprocity and contributes to a shared identity and group solidarity (See Table 3).

**Table 3:** Context-Mechnism-Outcome Supporting Self-organization and Emergent Collective Action

**Organizational strategy**	**CMO Configuration**	**Organization **
Supporting self-organization and emergent collective action	Small group activities (during which girls exchange experiences) that are embedded in the safety of the organizational environment (C) enables girls to identify problems they commonly face, support each other and build group solidarity and group identity (O). Joint sense-making of their experiences (M) within the safety of the organizational environment (C) enables the girls to individually and collectively reflect on their relations with peers, their families and on neighbourhood issues, including on their role within the neighbourhood community, generates an awareness of having a common purpose for collective action in the neighbourhood space (O). Identification of problems, reflection in group, a strengthened group identity and solidarity, and awareness of their rights contributes to girls exercising stronger individual and collective agency (O) and making empowered decisions about their lives and relationships, and their environment (O) as they are able to analyse the world with a gender lens and to identify disempowering structures requiring collective action for transformative change (M).	O1 and O2

Abbreviation: CMO, context-mechnism-outcome.

 “*When we talk about empowerment and services and building relationships, it is also done through these groups. As an individual, I will not be able to support anyone, but when there is a group, even if one person helps in a small way, a lot of support is provided to an individual, and the feeling of support and solidarity comes when people sit together and discuss. If we give lecture to a person individually, then the person may not understand, but if we sit with the group together and talk about it, through our own stories and anecdotes, then more will come out of it” *(O1, staff).

 Among the members of O2, the sense of solidarity was particularly strong. The girls faced great hurdles to play football. Initially, girls would literally have to stand their ground in public spaces and refuse to give way to boys, despite being stared down at or receiving verbal abuse. In a context where girls’ freedom is severely restricted and where they are monitored by family members (including their access to a mobile phone), such exchange of experience requires a safe space, which both O1 and O2 provide. In both neighbourhoods, there are no public spaces to “hang out” and girls are supposed to go directly home (and stay there) after school.

 The organizations and the girls recognize the power of a group identity in bringing about change and in catalyzing collective action. The small groups are not merely meant to be spaces for individual growth and solidarity, but a vehicle for social change. For instance, a member of O1 said:

 “*Our way of working on women’s empowerment is by making groups. We give more importance to collective work. If there are no services, if one person is speaking out, then services may not (be) provided. But if 10 people are saying the same thing, then government is alerted, and the service providers also wake up and people are then able to have their services. What we do is we make small groups, (…); we have quite empowered the groups” *(O1, staff member).

####  Context-Mechnism-Outcome Documenting “Lived Realities” and Engagement in Local Governance

 One of the objectives of the organizations is to stimulate their members to become active citizens, who engage in solving problems in the neighbourhood or contribute to its development:

 “*Similarly, football also is a means of making oneself visible, connecting with one’s neighbourhood, thinking about gender and space in a more actionable way. … With football, we also help them identify issues in the neighbourhood, solve them, and become better citizens” *(O2, staff member).

 To do so, O1 actively supports women and girls groups in systematically documenting their lived realities, through, for instance, safety audits. The latter involves an assessment of the safety of public spaces in the neighbourhood. In “youth-friendly SRHR audits,” the girls play-acted as mystery clients at public hospitals and first line health facilities to examine the responsiveness of health services to adolescents:

 “*The one I went for was to get services for STDs [sexually transmitted diseases]. I played an unmarried woman who had sex with her boyfriend, and who wants to get checked. The doctor was very strange, they did not behave well (…). It was pathetic. Just to do a test, it took (…) three days. They sent me from here to there. The doctor was so rude” *(O1, girl leader speaking of her experience while conducting the mystery client audit).

 O2 aims at strengthening the voice of youth through organising writing and journaling classes which enable the girls to be informed citizens, and to engage in discourse related to public issues affecting their lives. The girls produced a youth manifesto ahead of local elections demanding change on a range of neighbourhood issues. They also contribute to a newsletter, which O2 distributes to local community leaders, elected representatives and policy-makers. O2 further organises activities improving the neighbourhood (such as tree planting drives, wall painting events, distribution of relief rations during the COVID-19 lockdown) which gives the adolescents a sense of purpose and “a sense of place,” as agents of change in the neighbourhood.

 Finally, both organizations want to transform the thinking on gender roles in the communities. To this end, they work a lot on alliance-building. Both have tapped into the resources of other organizations. O1, for instance, established a local NGO forum that includes the Accredited Social Health Activist, Anganwadi workers and the community development officers, and organizes events together with other civil society organizations. The pooling of efforts attracts policy-makers’ attention. Both organizations use the media to amplify messages. In the case of O2, the media has played a major role in legitimizing O2’s collective action strategies in relation to adolescent girls’ voice and access to public space for the local community (See Table 4).

**Table 4:** Context-Mechnism-Outcome Configuration Documenting “Lived Realities” and Engagement in Local Governance

**Organizational strategy**	**CMO Configuration**	**Organization **
Documenting ground realities and engagement in local govern-ance	Documenting the neighbourhood’s problems and addressing these through adolescent collective action increases visibility (M) for adolescent health issues on the one hand and legitimacy and goodwill (M) in the community on the other hand. Community support for adolescent issues might raise the chance of adolescent needs being addressed by policy-makers (O) and change in thinking on gender roles (O). The organizations, in their activities, aim to strengthen adolescents’ voice to be informed citizens and change agents (M) so they can engage in local governance (O), enhancing their sense of place (O). Both organizations pool efforts with other organizations and engage in alliance-building, amongst others, and use the media to be more visible (M) to policy-makers so that they can weigh in on local policy-making (O).	O1 and O2

Abbreviation: CMO, context-mechnism-outcome.

###  Step 3: Context and Mechanism Analysis Using the Structure-Agency-Culture Roadmap 

 We follow here the SAC roadmap as presented in Van Belle et al^16^ (See Box 1).

**Box 1. Structure-Agency-Culture Roadmap^a^**

1: Transformative agency: Identify actors’ practices with change potential

2: Proximal context: Demarcate the context of action

3: Distal context: Analyse the structural and cultural conditions predating the context of action

4: Agency: How do actors’ practices influence the behaviour of power holders?

5: Causal mechanisms: Identify causal mechanisms underlying actors’ practices with change potential

6: Potential for sustained change: Explore potential for sustained change

----------------

^a^ Adapted from Van Belle et al.^16^

####  Transformative Agency 

 In this step, the actors’ practices that possess transformative potential are identified. Both O1 and O2 invest time in developing the critical consciousness of the adolescent girls, which is a precursor to their individual and collective agency. To achieve sustained change (by way of collective action), adolescent girls must first be capable to identify issues, reflect on how to best address these, and most importantly, see themselves in the role of change agents.

 The strategies and practices of the organizations differ, determined by their organizational development – O1 being a much older organization than O2.

- O1 built an intergenerational model of self-organization and uses proven tools (such as audits) to demonstrate the gaps in service delivery to policy-makers. This intergenerational model evolved into a “family” of women groups, with children and grandchildren of (the original) mothers becoming members of the organization.
- O2 is a young organization, rooted in the neighbourhood experiences of the organizations’ leaders. It uses place-based strategies to effect immediate change for the adolescent girls’ members, their families and the neighbourhood communities. O2 invests considerable time in working with the parents: in the N2, the mothers are not organized and O2 organizes events like picnics to get them on board.

####  Proximal Context 

 This step consists of demarcating the context of action from the structural and material conditions predating it. The context of action consists of the practices of stakeholders and the systems informing these. We identified the local governance system, the informality of the neighbourhoods and the safety of public spaces as the main elements.

- The organizations’ strategies and actions to promote accountability towards adolescents are circumscribed by the local governance system, consisting of relationships and practices, rules and norms of local power holders, such as community leaders, influential people bridging networks and administrators, and policy-makers.
- The neighbourhoods under study straddle the formal-informal divide, and the organizations need to support adolescents how to best navigate and engage with actors in this particular, quasi-informal realm.^26^ An example is O1 conducting mystery client visits to facilities to demonstrate the lack of adapted ASRH service delivery (including lack of access to contraceptives for non-married adolescents).
- Access to and safety of public spaces for girls is the third element. O2 let adolescent girls play football in a public space, defying gender norms. It had to use a more private venue because the girls did not feel comfortable. It should be noted that in both neighbourhoods, the freedom or autonomy of the girls is restricted. Older brothers often control the mobility of their sisters.

####  Distal Context

 In this step, the structural and cultural conditions predating the context of action are identified. Van Belle et alidentified quasi-permanent resource scarcity and systems of social exclusion and as structural conditions common to the Indian study sites. A system of intersecting layers of exclusion based upon caste, religion, ethnicity, internal migration status and socio-economic status marks the quasi-informal neighbourhoods. This contributes to the marginalization of its communities and restricted access to public services and citizenship entitlements. Traditional gender and socio-cultural norms restrict participation of adolescent girls in public life, including the exercise of their civic rights. The organizations’ actions are also embedded in the history of the women’s movement and their resistance to prevailing gender norms in India.^27^ Finally, their actions are based on the principles enshrined in the Indian Constitution.

####  Agency

 In this step, the interactions between those vying for change and the local power holders are examined. How do the practices of actors seeking change influence the behaviour of power holders? Changing the practices of power holders expands the potential for sustained social change.

 Both O1 and O2 are engaging with local powerholders and consider their legitimacy as key:

 “*When you go with the support of community members, you automatically become a representative of them. Only then, pressure can be created and I believe this is what the job of civil society is - to build the pressure and continue questioning” *(O2, staff).

 Both organizations consider the adolescent girls’ activities to community development (such as tree planting, painting walls, ration distribution during the lockdowns) as central to change, as it makes the community leaders see the groups in a positive light. More importantly, this stimulates the girls’ sense of place, of connection with and belonging to the neighbourhood.

 O1 and O2 use different strategies to change perceptions at different levels. O1 was set up during the Indian 1970s women’s movement and, led primarily by urban middle class women, organized widespread sit-ins and demonstrations against sexual violence. Activists became leaders of contemporary women’s civil society organizations. In the early 1980s, organizations shifted from national level legal action to local self-organization and mobilization.^27^ It has a long history of alliance-building with NGOs at the city and district policy-making level, beyond the intervention sites. Being part of NGO platforms has expanded its influence and visibility.

 O2 was set up recently by local women leaders. Its actions are more localized, and considerable attention goes to individual empowerment, helping adolescent girls fulfil their personal aspirations besides aiming at group empowerment. O2 currently organizes events to draw in the communities and change their perception on what girls can and cannot do. This has attracted the attention of sub-national policy-makers, who wish to be associated with O2’s events. O2 also wants to strengthen girls’ participation in civic activities – see for instance the youth manifesto discussed above.

####  Causal Mechanisms

 In this step,we explore the causal mechanisms that underlie the actors’ practices with change potential. In this study, group solidarity, the trust of parents in the organizations and legitimacy in the community emerged as important mechanisms.

- Group solidarity is an important precursor mechanism to the mobilization of adolescent girls into collectives and the creation of women’s organizations. The group solidarity of any social movement (such as the women’s movement) is grounded in a shared history of oppression.^28^
- Trust of parents in the organizations and its leadership is a necessary, but insufficient condition for girls to be able to participate in the activities. Parents and older brothers play a gatekeeper role. Other authors, such as Game^29^ found that trust of parents and their buy-in are key to the participation of marginalized adolescent girls in interventions aiming at empowerment through sports.
- To enable their activities, organizations seek legitimacy in the community by for instance organizing events in public space.^30^ They require the additional support of the broader community, including community leaders and other organizations as religious leaders did not always support the organization’s actions.

Our IPT drew from both social science theories and empirical evidence to identify the causal mechanisms, and specifically built upon “agonism” (following the philosophy of agonistic pluralism Chantal Mouffe), acknowledging that groups will have different views which can be expressed. One can however build on constructive conflict to join forces in collective action, and “consensus” (following the philosophy of Habermas in his “theory of communicative action”), groups can come together to form a consensus on what to do based on rational deliberation. We found that both organizations use strategies based on agonism and consensus- building strategies.

 Currently, O1 and O2 use consensus-oriented strategies. For example, they “nudge” the communities towards support, appealing for place-based solidarity in a segregated neighbourhood.^31^ For example, to advocate for a children’s playground, they collected signatures from the mothers or other groups. Another tactic is collaboration with other organizations: O2 set up a formal collaboration with other NGOs.Agonistic strategies include audits and mystery client visits, which serve to demonstrate the lack of adapted ASRH services to local government actors and administrators. O1 initially relied on demonstrations and other agonistic actions, but evolved towards more consensus-oriented strategies in its engagement with state and national level actors, such as the participation in committees, NGO policy forums and networks. This occurred as the policy landscape vis-à-vis gender equality evolved, however protests and demonstrations are still considered a part of their repertoire.

 The organizations also use the media as an instrument to make issues salient for local government actors. O2, for instance, created media visibility through to the perceived novelty of its actions, which then attracted the attention of policy-makers seeking to be identified with these actions.

####  Pathways Towards Sustained Change

 The analysis ends with the exploration of the potential for sustained change, understood as the change in structural and cultural conditions.

 We found that the organizations’ strategies towards adolescents co-evolved with their own organizational development and the interactions with the adolescents and communities. They are embedded in the local and national policy context and shaped by their relationships with authorities at multiple levels. We found they have different pathways towards sustained change, as the scale (or remit) of their action differs. O1’s strategy plays out at different levels simultaneously. Its ability to keep up continuous pressure has proven crucial. They stimulate self-organization of local women’s groups based on an intergenerational model that has given rise to a “family” of women groups. O2 is primarily invested in supporting the community it works in and it strengthens the individual and collective agency of adolescent girls. It gradually expanded its actions in the neighbourhood, starting off with football and now organizing action for a public playground. It adapts it actions in line with the local policy context. It is too early to say if their actions will lead to sustained change. O2 also engages with policy-makers at sub-national and national level and builds alliances with NGOs to influence policy. As a result, their potential to achieve sustained change is greater. The SAC analysis sheds light on the organizations’ historical co-evolvement with the environment and provides a forward-looking perspective to sustained social change.

###  Step 4: Formulation of the Refined Programme Theory

 The final phase of a realist study is the refinement of the IPT.

 Our analysis shows that organizations that aim at strengthening community engagement and self-organization within communities to demand accountability from local government can use both consensus-oriented and agonistic strategies. We could not confirm that the organizations’ strategies *ultimately* led to improved adolescent health services. However, we found that gender-based group solidarity and critical awareness of citizenship gave rise to emergent collective action. The latter can nudge local non-state actors to jointly demand accountability from state actors, by way of consensus-oriented and agonistic strategies and bridging different positions, roles and interests at neighbourhood level.

 We formulated our refined programme theory as follows:

 “In a structural context with a social order where mainstream institutions and processes reproduce social exclusion founded on ethnicity, gender, religion or on other differences, and a quasi-permanent resource scarcity, and in a cultural context in which patriarchal norms intersect with religion, ethnicity, citizenship status and socio-economic status, adolescent girls engage in individual and collective agency (M) to achieve better adolescent health and well-being outcomes (O) supported by grassroots organizational strategies. The latter include (1) mobilizing adolescent girls and young women, (2) raising critical consciousness, (3) supporting self-organization and emergent collective action, and (4) documenting ground realities and engagement in local governance. Strategies that are founded upon mechanisms of trust-building in the community and with parents enable necessary support, foster a group identity and provide a safe space for peer exchange. They enhance reflexivity, self-efficacy and a sense of place among adolescent girls. This enables emergent collective action within the neighbourhood and dependent on the context, which in turn contribute to build consensus and enforce accountability with local and sub-national governance actors.”

 In this study, we set out to test a hypothesis (formulated as an IPT) of how grassroots organizations shape accountability practices towards adolescent health through collective action and the engagement of civil society organizations with state actors in marginalized urban neighbourhoods. Our IPT made use of Kapoor^32^ to distinguish between two main “either/or” strategies: consensus-oriented strategies (aligned with Habermas’ theory on communicative action) or agonistic pluralism (aligned with the philosophy of Mouffe) applicable in the context of under-resourced settings in low- and middle-income country.^16^ Our hypothesis was that organizations will simultaneously engage in consensus-oriented and agonistic strategies with state actors in their efforts to achieve social change.

## Discussion

 We found that the relationships between governance actors (between local governance actors and with governance actors at other levels) in Indian cities form a complex web. First, grassroots organizations supporting adolescent health need the local community to stand with them, as adolescent girls (and their needs) might not provide sufficient weight to influence local decision-making on their own. Second, consensus-oriented actions between government and civil society and agonistic actions from the grassroots demanding better accountability from the government, co-exist. We found indeed that organizations use both types of strategy simultaneously, showing nimbleness or agility in their efforts to shape local public policy decisions. Third, policy-makers seek out to be associated with positive collective action, blurring boundaries between “shown intent” and “actual action.” We found policy-makers wanting to be associated with highly visible, “positive” civil society events, such as adolescent girls playing football, which can be leveraged, but consistent negotiation is required to concretely deliver on adolescent and young peoples’ needs.

 International organizations have recently hailed the idea of youth engagement in health policy-making, which has contributed to an increase in youth co-creation initiatives. Yet, the latter may underestimate the power dynamics in the local governance ecosystem and the strength of countervailing norms.^33^ Adolescents may be cancelled out of decision-making by power holders who either claim that youth are not interested or that they merely echo the opinions of those around them.^34^ Perhaps a lesser heard argument is Jessica Taft’s, who explored the political lives of Latino girls: she claims that adolescent girls, more than boys, are being tasked with solving the community’s problems. They are considered as the “girl saviours” of the entire community.^35,36^

 While there is merit to these arguments, we find that organizations such as those described in this paper, provide a crucible for youth and adolescent empowerment that is not necessarily directed towards vertical development goals, but rather bottom-up empowerment and leadership building, which can push-back against tokenistic involvement of youth in policy and programmes. The implications for policy-makers then, is that state-driven efforts seeking to strengthen ASRH, while ambitious, need the work of community-based organizations to create conditions in which adolescent and young people can learn to exercise agency, make informed decisions about their own health, and take collective action to assert their rights. Community-based work over several decades requires investment, which tends to be undervalued when it does not produce direct health outcomes, and may even be seen as inconvenient for policy-makers. However, without its contributions, health programs can end up being perfunctory and ultimately fail. The strength of these organizations is that they function independently outside the health system, and they should not be used instrumentally by health systems to expand reach of their programs. Rather, they should be treated as sensitive and valuable agents of change in their own right, whose work can bring about social change which is important for health and other systems.

 Methodologically, the integration of the SAC framework in the analysis has put the role of collective and individual agency in the organizational strategies of grassroots organizations front and centre. Their role in effectuating durable social change is at times underplayed in urban studies and community-based SRHR interventions.^37^ Following Archers’ SAC analytical framework, we considered causal mechanisms to be historically grounded in practices preceding current ones, and we adopted a view on “context” that includes a longer-term temporal dimension.As mentioned in the introduction, Archer’s framework introduces a longer temporal perspective in RE and engages with the path dependence of grassroots organizational action, perhaps leading to more global level policy recognition of their salience in achieving social change.^6^

###  Study Limitations

 The COVID-19 pandemic and the control measures hindered the IDIs and FGDs because of travel and contact restrictions. Local government authorities and organizations were busy managing the pandemic. Yet, we managed to obtain adequate data through flexibly reorganising the interviews and FGDs.

 The embeddedness of the study in organizations is both a limitation and strength of the study. It could be argued that there is a bias in the selection of participants and in the interpretation of data, however we believe that the trust and shared learning agenda of the organizations and the research team ensured that only “success stories” were not shared.

 It can be argued that we used theories originating in high-income countries. This reflects the lack of theoretical and empirical literature on the themes of this study. However, we also made use of authors such as Kapoor^32^ who verified the applicability of the Habermas-Mouffe theoretical debate in under-resourced settings of low- and middle-income country. The realist approach offers the advantage of testing explicit hypotheses through empirical research, tasking the researcher to test these in the study settings. This allowed to see in how far theories from whichever origin allow for causal explanations of outcomes and processes observed in the study sites.^38^

## Conclusions

 We tested and refined an IPT on adolescent agency and accountability in urban marginalized neighbourhoods. We carried out a case study of two organizations and obtained a better understanding of the mechanisms underlying their interventions. We also demonstrated the applicability of methodological guidance to integrate Archer’s SAC framework in RE and found that this helps to better analyse the interaction between agency and structure, to look at collective and individual agency, and to better understand temporal dynamics.

## Acknowledgements

 We would like to thank the organizations, and especially their leadership, and the adolescent girls, for accommodating this research study. Unfortunately, we cannot name the organizations, as we uphold the principles of protecting the anonymity and confidentiality of all interviewees.

## Disclosure of artificial intelligence (AI) use

 Not applicable.

## Ethical issues

 The study has been approved by the Institutional Review Board of the Institute of Tropical Medicine, Antwerp, Belgium.

## Conflicts of interest

 Sara Van Belle is funded by a senior postdoctoral fellowship awarded by the Flemish-Belgian Fund for Scientific Research (Grant number: 1221821 N).

## Data availability statement

 Focus group guides are available as Supplementary files 1-4. Other data cannot be shared due to the ethics and informed consent and assent procedures which ensured fully anonymized data to protect the confidentiality of the study participants.

## Supplementary files


Supplementary file 1. Coding Tree.

Supplementary file 2. Topic Guide 1 In-depth Interview With Organization Staff.

Supplementary file 3. Topic Guide 2 In-depth Interview With Formal and Informal Governance Actors at Local and State Level.

Supplementary file 4. Topic Guide 3 FGD/Interview Facilitation With Adolescents and Young Women.
