# Supplementary material for: Deepening Context in Realist Evaluation: Exploring Adolescent Agency in Sexual and Reproductive Health in Urban India
Source: Int J Health Policy Manag. 2026 Jun 10;15:8837. doi: 10.34172/ijhpm.8837 (PMC13729708; doi:10.34172/ijhpm.8837)
Supplement: Supplementary file 1 [file ijhpm-15-8837-s001.pdf]

Article title: Deepening Context in Realist Evaluation: Exploring Adolescent Agency in Sexual and Reproductive Health in Urban India

Journal name: International Journal of Health Policy and Management (IJHPM)

Authors' information: Sara Van Belle[1]*, Sana Contractor[2], Sandhya Gautam[3]

[1]Department of Public Health, Institute of Tropical Medicine, Antwerp, Belgium.

[2]Independent Consultant, Mumbai, India.

[3]Seher - Center for Health and Social Justice, New Delhi, India.

***Correspondence to:** Sara Van Belle; Email: svanbelle@itg.be

Citation: Van Belle S, Contractor S, Gautam S. Deepening context in realist evaluation: exploring adolescent agency in sexual and reproductive health in urban India. Int J Health Policy Manag. 2026;15:8837. doi:10.34172/ijhpm.8837

Supplementary file 1. Coding Tree

CODING TREE

- - Information about the neighbourhood
    - History of settlement
    - Social Groups
    - Work/employment
    - Civic facilities
    - Perception of discrimination
- Gender based restrictions
    - Education
    - Household work
    - Mobility
    - Access to public spaces
    - Relationships and marriage
    - Coercion and violence
- Health and health services
    - Major SRH problems
    - SRH facilities
    - Problems with health facilities
    - Impact of COVID 19 pandemic
- Organization's history
- Organization's Strategies
    - Mobilization and building of a collective
    - Consciousness raising
    - Documenting, generating evidence and articulating realities
    - Alliance building
    - Advocacy with the state
- Outcomes
    - Individual
    - Relationships
    - Relationship to state
    - Changes in public services
