# Supplementary material for: Deepening Context in Realist Evaluation: Exploring Adolescent Agency in Sexual and Reproductive Health in Urban India
Source: Int J Health Policy Manag. 2026 Jun 10;15:8837. doi: 10.34172/ijhpm.8837 (PMC13729708; doi:10.34172/ijhpm.8837)
Supplement: Supplementary file 2 [file ijhpm-15-8837-s002.pdf]

Article title: Deepening Context in Realist Evaluation: Exploring Adolescent Agency in Sexual and Reproductive Health in Urban India

Journal name: International Journal of Health Policy and Management (IJHPM)

Authors' information: Sara Van Belle[1]*, Sana Contractor[2], Sandhya Gautam[3]

[1]Department of Public Health, Institute of Tropical Medicine, Antwerp, Belgium.

[2]Independent Consultant, Mumbai, India.

[3]Seher - Center for Health and Social Justice, New Delhi, India.

***Correspondence to:** Sara Van Belle; Email: svanbelle@itg.be

Citation: Van Belle S, Contractor S, Gautam S. Deepening context in realist evaluation: exploring adolescent agency in sexual and reproductive health in urban India. Int J Health Policy Manag. 2026;15:8837. doi:10.34172/ijhpm.8837

Supplementary file 2. Topic Guide 1 In-depth Interview With Organization Staff

Topic Guide 1 In-depth Interview with organization staff

Profiles: director of organization; organization’s founder; staff working with adolescents

Interview type: in-depth interview

Themes: accountability strategies of the grassroots organization; accountability ecosystem - formal and informal accountability relationships; socio-political context and citizenship; social exclusion; local collective action & solidarity

Elements	Actions / Questions	Codes
Arrival	Settling in	
Introduction	Intro interview (objectives of the study)<br>Written informed consent procedure<br>Ask permission to audio-record and to take notes	
Equipment check	Check the audio conditions and if tape recorder is functioning	
General part	Q: Could you please describe to me your responsibilities as …?<br>Q: How long have you been working in…?<br>Q: How long have you been working in this location?	
**Themes**	**Transition to key questions**	
Organisational activities, mission, vision<br><br>Organisational history<br><br>Socio-political context<br><br>Organisational evolution	Q : Can you describe the activities of the organization?<br>Q: What is its current mission and vision? probe: what you want to change, or how you want to contribute to change?<br>Q: Can you describe the main strategies through which you want to achieve this?<br>Q: how would you describe your target group?<br>Q: When was the organization founded? Why?<br>Q: Can you describe the context of that time? Probe: the socio-political context and the situation the target group was in at the time<br>Q: How has the organization evolved over the years? Probe: has kit changed or expanded its activities, its mission?<br>Q: How does the organization attract funding? Probe: expansion due to more funding available (local -national-foreign)<br>Q: How would you describe the organization today? Probe: professionalization over the years, still recent and finding its way,…	
Target group, social exclusion, vulnerability	Q: Can you describe the situation of the target group?<br>Q: Would you consider these adolescent girls / young women a vulnerable group?<br>Q: What is the meaning of vulnerability in this context? Probe: relation to being able to use public space; access to services; stigma / social discrimination; ability to exercise citizenship.	

ASRHR / sanitation/safety public space<br>/ coping / empowerment	Q: What is the situation here in the district / region regarding adolescent/youth sexual and reproductive health? What are the main issues? Probe: early marriage, sexual violence / GBV,<br>Q: How are health services organized (public, subcontracted to INGOs, etc)?<br>Q: What are the issues with health service delivery for adolescents and youth? (probe: access in terms of acceptability, availability, …)<br>Q: How would you describe the youth-friendliness of services? Probe: Are they being responsive to their needs? How could they improve their responsiveness?<br>Q: What is the situation in terms of sanitation facilities?<br>Q: In your view, can adolescents freely congregate in this neighbourhood? If not, what are the risks they encounter?<br>Q: What would be coping strategies of adolescents and youth to deal with lack of health services / sub-optimal sanitation/ safety public space?<br>Q : What has been the impact of covid-19 on this neighbourhood and the situation of adolescent girls?	
Accountability strategies	Q: Can you describe what accountability means to you?<br>Q: How would your activities contribute to empower adolescents and youth to claim accountability?<br>Q: How does the organization claim / enforce accountability on their behalf? To whom?	
Other NGOs/ CBOs involved; stakeholders; emergent collective action; social capital/solidarity	Q: Are they other organizations that you work with or other individuals (community leaders, religious leaders) you work with that help you in your work? Probe: whom you could consider allies?<br>Q: Can you give an example of actions you mounted together? What was the result of this action?<br>Q: On what was the solidarity based between the actors? Probe: do you think there is a sense of solidarity in the neighbourhood? On what is this based?	
Relationships with formal governance actors (state, municipal corporation) & accountability practices	Q: Could you describe your relationship with the municipal corporation? With whom / which department are you in contact? Do they invite you to meetings (which ones)? Are you asked to report or to inform them? Have you worked together on joint activities? Would you say they are concerned about the situation of adolescents? Adolescents in this neighbourhood? Is there a specific policy or programme? Are you involved in annual planning exercises?<br>Q: Could you describe your relationship with the state authorities? (similar questioning as with municipal authorities with focus on policy development and planning re the state's competences)<br>Q: Do you work together with the schools? With the health facilities?<br>Q: Who, in your view, represents the voice of adolescents and youth vis à vis the authorities? How would you ensure they have a voice?	

**Relationship informal governance actors & accountability practices (community leaders, religious leaders)**	Q: How would you describe your relationship with local leaders? With whom do you work regularly? Do you mount actions together? Are you invited to their meetings?	
**Responsiveness / accountability most vulnerable**	Q: How do you reach the most vulnerable? Those who do not come to the organisation's activities? How in your view can you increase accountability towards this group?<br>Q: If you would consult with community representatives or community based organizations, would they, in your view, reflect the views of the groups that you do not reach?<br>Q: Have there been discussions with INGOs (on how to reach the ones who are most in need and who do not make use of the services? Have there been incidents you know of that sparked these discussions?	
**Ensuring accountability / leadership ("meta governance") / power**	Q: If you look at all actors doing activities for adolescents, which actor would be best placed to ensure that all actors are accountable (to adolescents)?<br>Q: Are there any accountability guidelines from the State / municipal corporation has to adhere to? For example, is there a mechanism in place to handle service user complaints?<br>Q: Does the state / municipal corporation have a mechanism in place to allow a citizens' representation to have its say in how to improve services? Who would represent the voice of adolescents? Who would represent the most vulnerable groups in the community?<br>Q: What would be the mechanism to enforce accountability towards the adolescent girls of this neighbourhood?	
**Announce the end of the interview**	**'A last question (…)'**	
**Change**	Q: If you were given a budget to improve ASRH accountability, what would you prioritize?<br>Q: how would you see the organization's future?<br>Q: how do you see adolescent accountability and empowerment strategies involving in terms of access to digital technologies probe: many adolescents have a smartphone…	
**After the interview**	**Word of thanks. Offer the possibility to withdraw from the study. Explain how the interview will be used.**	
