# Supplementary material for: Deepening Context in Realist Evaluation: Exploring Adolescent Agency in Sexual and Reproductive Health in Urban India
Source: Int J Health Policy Manag. 2026 Jun 10;15:8837. doi: 10.34172/ijhpm.8837 (PMC13729708; doi:10.34172/ijhpm.8837)
Supplement: Supplementary file 4 [file ijhpm-15-8837-s004.pdf]

Article title: Deepening Context in Realist Evaluation: Exploring Adolescent Agency in Sexual and Reproductive Health in Urban India

Journal name: International Journal of Health Policy and Management (IJHPM)

Authors' information: Sara Van Belle[1]*, Sana Contractor[2], Sandhya Gautam[3]

[1]Department of Public Health, Institute of Tropical Medicine, Antwerp, Belgium.

[2]Independent Consultant, Mumbai, India.

[3]Seher - Center for Health and Social Justice, New Delhi, India.

***Correspondence to:** Sara Van Belle; Email: svanbelle@itg.be

Citation: Van Belle S, Contractor S, Gautam S. Deepening context in realist evaluation: exploring adolescent agency in sexual and reproductive health in urban India. Int J Health Policy Manag. 2026;15:8837. doi:10.34172/ijhpm.8837

Supplementary file 4. Topic Guide 3 FGD/Interview Facilitation With Adolescents and Young Women

Topic Guide 3 FGD/Interview facilitation with adolescents and young women		
Profile: Adolescents and young women who have been selected by the grassroots organization, with written informed consent and assent, preferably those that were part of a focus group discussion<br>Type: focus group discussion and in-depth interview; in-dept interviews are for discussing more sensitive or personal issues related to ASRH.<br>Remind respondent that the interview will remain confidential and its results anonymized, that no one will be able to trace back comments, and explain the opt out rules.		
Elements	Actions / Questions	Codes
Arrival	Settling in	
Introduction	Intro interview (objectives of the study)<br>Written informed consent procedure<br>Ask permission to audio-record and to take notes	
Equipment check	Check the audio conditions and if tape recorder is functioning	
General part	Q: Could you please describe to me your responsibilities?	
Themes	Transition to key questions	
Health services access / health seeking behaviour	FGD/Q: If you have a health issue related to SRH (e.g. an RTI or thrush; need for FP), where would you go to to find a provider with whom you are comfortable (in terms of confidentiality, privacy…)? Is there a provider close by or long distance from your home (or prefer long distance because of fear of disclosure)? Would you go public / private; prefer it to be a man/ woman? (probe: attitudes providers)	
Sanitation facilities	FGD : Can you describe the situation in terms of sanitation? FGD lead / co-PI use examples encountered.<br>Q: What could be done to improve the situation?<br>Q: How would you / the organization ask attention for this problem? What would draw the attention of policy-makers? (e.g. municipal councillors, administration,…)	
Use of public space / safety / transport	FGD: Draw a map of the neighbourhood, of safe and unsafe spaces, and where you come together / meet up with friends (protected space)<br>Q: How long do you have to travel to go to school? Do you have to travel on your own? Do you meet up with friends to travel to school and back home?<br>Q: How long do you have to travel to go to a health service facility where you would feel comfortable seeking help or advice on ASRH issues?<br>Q: Do you use public transport to go to school / shops /health facility? Do you feel safe on public transport?<br>Q: Are there certain hours that it is not safe to be on the streets (e.g. after sundown)?<br>Q: What hours do your parents tell you you have to be inside?	

Seeking help / coping strategies	FGD: Who would you go to if you need advice on an ASRH issue (boyfriend trouble, a girl you know thinks she's pregnant, menstruation problem); a friend, family, teacher, internet, organization, adolescent-friendly service provider? (Discuss a top 3 of people and organizations/institutions)<br>Q (in-depth interview): referring to FGD, ask for stories / narratives on incidents	
Grassroots organization activities / empowerment	FGD/Q: Since when have you been with the organization? How did you come to be involved? What attracted you? What are the activities from the organization you have participate in? What are your favourite things? What do you think this organization does particularly well (probe: what organizations do not do)?<br>FGD/Q: What are some of the changes that have come about in your life due to association with the organization? Probe – individual, relationships, community<br>What kinds of activities have you done as a group with the organisation? Probe – collective campaigns, demands, protests	
Grassroots organization/ accountability strategies	FGD/Q: How does this organization help the cause of young women in your opinion? Probe: does it represent you vis à vis whom (policy makers)<br>Q: How does the organization strengthen your voice in your view?	
Announce the end of the interview / FGD	'A last question (…)'	
Change	FGD/Q: If you were in charge, what would you go for in terms of improving the situation for adolescent girls? Probe: what would you advice the people from the municipal corporation?<br>FGD/Q: How would you want the organization to help with that?	
After the interview	Word of thanks. Offer the possibility to withdraw from the study. Explain how the interview will be used.	
